# Comparative Efficacy of Mitchell’s and Benson’s Relaxation Techniques in Alleviating Pain and Improving Quality of Life Among Patients With Premenstrual Syndrome: A Randomized Controlled Trial

**DOI:** 10.7759/cureus.43877

**Published:** 2023-08-21

**Authors:** Anushka Raipure, Shubhangi Patil

**Affiliations:** 1 Department of Community Health Physiotherapy, Ravi Nair Physiotherapy College, Datta Meghe Institute of Higher Education & Research (DU), Wardha, IND

**Keywords:** relaxation techniques, quality of life, numerical pain rating scale, premenstrual syndrome questionnaire, premenstrual syndrome, benson’s relaxation technique, mitchell’s relaxation technique

## Abstract

Background

Most women of reproductive age suffer physical discomfort or distress in the weeks before menstruation. Even though symptoms are typically not severe enough to impede daily activities seriously, they occasionally can. Physical problems like breast discomfort and bloating can also be a problem. The most irritating symptoms are those that affect mood and behaviour. Women of reproductive age experience premenstrual syndrome frequently, necessitating study into non-pharmacological methods for symptom reduction.

Methodology

The objective of the study was to evaluate and compare the clinical efficacy of Benson's relaxation technique to Laura Mitchell's physiological approach in patients with premenstrual syndrome. Study design was comparative parallel experimental study with patient-reported questionnaire data (online) collected before and post-intervention in tertiary hospital setting. Participants were 70 adult females aged 18 to 35 with premenstrual syndrome. Patients were administered Benson’s relaxation technique once a day for a month versus Mitchell’s relaxation technique once a day for a month. Both techniques were first taught for one session followed by home program to be performed by patients. The premenstrual syndrome questionnaire and a numerical pain rating scale were used to quantify premenstrual symptoms pre and post-intervention.

Results

The result revealed significant (p<0.01) improvement in premenstrual symptoms in both groups following the intervention. However, Bensons' relaxation technique was found to be more significant while alleviating the premenstrual symptoms.

Conclusions

When it comes to lowering the intensity of premenstrual syndrome in young people, Benson's relaxation method is superior to Mitchell's. Both approaches should be entrenched as a regular practice and can be employed on patients to improve their menstrual well-being.

## Introduction

Women's reproductive cycles are cyclical, leading to significant impacts on both physical and mental health. Premenstrual syndrome (PMS) is a psychosomatic condition closely linked to female reproductive function [1]. PMS encompasses a group of complex and interrelated symptoms with pathophysiological events that commence with ovulation [2]. It manifests through more than 300 behavioural, social, emotional, psychological, and physical symptoms, such as changes in appetite, weight gain, headaches, nausea, constipation, breast enlargement, soreness, anxiety, aggression, irritability, fatigue, and mood swings [3].

Despite the widespread prevalence (14.3%-74.4%) of PMS, only a limited number of treatment options are available [4]. The primary goal of PMS treatment is to alleviate both physical and mental symptoms. Many commonly prescribed medications prevent ovulation or affect neurotransmitter levels in the brain, such as serotonin, norepinephrine, and dopamine. Additionally, complementary or alternative medicines with distinct modes of action are utilized [5]. Due to the adverse side effects of pharmaceutical therapy and surgery, non-drug remedies have gained interest among professionals and women seeking alternatives [6]. Alternative treatments can empower patients and lead to positive outcomes. Education plays a significant role, with recommendations on managing weight, quitting smoking, reducing alcohol consumption, limiting salt intake, and incorporating exercise being crucial. Psychological approaches like behavioural therapy and relaxation training are also critical, as research in this field has demonstrated the benefits of relaxation [7].

Relaxation practices actively boost immune system performance, lessen depression, and enhance the quality of life. Furthermore, these practices effectively reduce tension and anxiety, contributing to improved psychological well-being [8]. Achieving a balance between the posterior and anterior hypothalamus can lead to a decrease in sympathetic nervous system activity and an increase in catecholamine secretion, resulting in muscle relaxation, controlled breathing, lower heart rate, reduced muscle spasms, and mitigated physiological side effects, anxiety, and fatigue [9]. However, despite these established benefits, further research is still needed to provide patients with the most effective relaxation programs [10].

For women experiencing premenstrual syndrome, various relaxation techniques can enhance their quality of life (QOL). This study centres around two specific methodologies, one of which is the physiological relaxation therapy developed by Laura Mitchell. Her approach has demonstrated remarkable success in enhancing immunological functions, alleviating depression, and improving QOL. The physiological concept of reciprocal inhibition forms the foundation of Mitchell's diaphragmatic breathing technique, wherein the activation of one muscle group acting on a joint corresponds with the relaxation of the opposing muscle group. Within her relaxation technique, Mitchell skillfully incorporates reciprocal relaxation, which involves moving one part of the body away from a tensioned area in the opposite direction before releasing it, thus promoting further relaxation [11].

Relaxation techniques mitigate unpleasant emotions, including fear, anxiety, anger, and despair, while controlling muscle tension. Benson's relaxation approach has been a practical and affordable stress-reduction method since 1970 [12]. Several relaxation techniques exist, but Herbert Benson's method from 1970 is particularly renowned for its effectiveness [13]. This relaxation method is simple and does not require special knowledge or tools. Moreover, it is suitable for patients of all ages. Previous research has demonstrated that Benson's muscular relaxation significantly reduces somatic contractions, effectively addressing stress's physiological and psychological effects [14]. The technique relies on four elements: creating a tranquil environment, adopting a relaxed position, maintaining focus (e.g., on one's breathing pattern), and actively relaxing muscles [15]. This approach actively decreases endogenous catecholamine levels and sympathetic nervous system activity, leading to muscle relaxation and reduced tension, anxiety, and depressive symptoms. Benson's relaxation method actively associates with an increase in self-esteem. By maintaining focus, individuals can actively control their breathing, lower their heart rate and blood pressure, and prevent numerous harmful physiological responses to stress [16].

Numerous studies have explored the efficacy of relaxation methods such as Jacobson, yoga, and pilates in addressing PMS. Though widely researched for various conditions, the Benson relaxation technique needs to be examined more in the context of PMS. This study, therefore, seeks to fill this research gap by examining the impact of Benson's relaxation technique, alongside Laura Mitchell's approach, on PMS symptoms. A systematic review by Jose et al. accentuates the efficacy of varied relaxation therapies. These encompass Progressive Muscle Relaxation (akin to Jacobson's method), Laura Mitchell's technique, and Benson's approach. The review further underscores the potency of uncomplicated relaxation modalities such as relaxation, yoga, aerobic activity, and massage in notably alleviating PMS symptoms [17]. However, comparative studies necessitate further investigation into the relative effectiveness of Laura Mitchell's relaxation technique and Benson's relaxation approach for treating PMS. Our study conducts a rigorous comparative analysis between Laura Mitchell's physiological process and Benson's relaxation method to address this gap. Our central objective is to discern which of these two techniques has superior efficacy in mitigating the multifaceted symptoms associated with premenstrual syndrome. Through this endeavor, we aspire to contribute substantively to the ongoing discourse on efficacious PMS interventions, offering valuable insights to healthcare practitioners and individuals grappling with this condition.

## Materials and methods

The study obtained clearance from the Datta Meghe Institute of Higher Education and Research institutional ethical committee (Ethical authorization number: DMIMS(DU)/IEC/2022/1024) before its commencement. Researchers conducted this comparative experimental study at a tertiary care hospital in Sawangi, Wardha, from September 2022 to February 2023. The participants in the study were adults with premenstrual syndrome, aged between 21 and 35 years. The study aimed to compare the clinical efficacy of Benson's and Mitchell's relaxation techniques as non-drug therapeutic interventions, adhering to the Consolidated Standards of Reporting Trials (CONSORT) requirements.

The number of patients participating in the study was determined using the Daniel formula for sample size, considering data from Tschudin et al.'s survey on the prevalence of premenstrual syndrome [18]. The researchers provided participants who completed the informed consent process with a link to complete the pre-intervention questionnaire. After completing the baseline questionnaire, the physiotherapist reviewed the techniques with the participants. Due to the nature of the study interventions, physiotherapists or research assistants were not feasible, however, the participants were unaware of the procedure they received. Upon completing the analysis, the researchers ensured they kept the trial statistician unaware of the allocation. Throughout the analysis phase, the researchers took measures to maintain the blinding of the statistician.

Participants eligible for inclusion in this study are female individuals aged between 16 and 50 years who experience regular menstrual cycles lasting three to eight days, with menstrual cycles spanning between 22 to 35 days. Potential participants must self-report premenstrual symptoms that are significantly interfering with their daily activities and are willing and capable of providing informed consent to participate. Individuals currently enrolled in any other clinical trial or those who are pregnant, breastfeeding, or experiencing irregular menstrual cycles outside the specified range will be excluded. Participants with a history of severe cardiovascular conditions or unstable cardiovascular status, known contraindications or medical conditions, mental health conditions that could impact participation or assessment, a history of substance abuse or dependence, inability to effectively understand or communicate in the study's language, individuals who have received treatment for premenstrual syndrome within the past three months, and those with any other medical or psychological condition that could potentially interfere with study participation or outcomes will also be excluded. Moreover, individuals with known diabetes, hypothyroidism, or polycystic ovary syndrome (PCOS) were excluded from the study. Participants falling under the categories of overweight or obesity based on their body mass index (BMI) were also excluded. These exclusion criteria aim to ensure a specific and well-defined study population and to control for potential confounding factors related to underlying medical conditions and lifestyle choices that could impact the evaluation of Benson's relaxation technique and Mitchell's physiological approach in alleviating premenstrual syndrome symptoms.

Data collection tool and technique

Seventy premenstrual syndrome patients were enrolled in the study and randomly assigned to one of two treatment groups. The physiotherapist screened the subjects based on the inclusion and exclusion criteria. Before participation, all subjects were informed about the study's purpose and provided written informed consent.

The subjects were divided into two groups, Group A and Group B, using random allocation. Pre- and post-intervention evaluations were conducted for all subjects using the Premenstrual Syndrome Questionnaire and the Numeric Pain Rating Scale. For 30 minutes, Group A received Laura Mitchell's progressive relaxation technique, while Group B received Benson's relaxation approach.

Researchers assessed the effects of the interventions by conducting pre- and post-intervention assessments of the subjects using the numeric pain rating scale and the premenstrual syndrome questionnaire. This rigorous design and evaluation process aims to determine the impact of Laura Mitchell's and Benson's relaxation techniques in alleviating symptoms of premenstrual syndrome and pain in the study participants.

Interventions

Group A: Laura Mitchell’s Physiological Relaxation Technique

The patient was positioned on a firm surface in a comfortable half-lying or supine position, with proper support from pillows and cushions to ensure complete relaxation and support for her body. The instructor gently covered the patient's eyes and mouth to create a calming environment. The instructor kept her voice soft and soothing during the relaxation training to promote a tranquil experience. Next, the instructor guided the patient to perform relaxation techniques, starting from her mouth and moving towards her lower extremities. The instructor asked her to gently force her tongue into her mouth and then draw her mouth downward. Subsequently, the instructor instructed the patient to press her head on the plinth, gently pulling her shoulders towards her feet and sliding her elbows sideways. Moving further, the instructor asked the patient to abduct and extend her fingers and thumbs. For patient's lower extremities, the instructor guided the patient to rotate her thighs outward and settle them comfortably. The instructor instructed the patient to position her knees comfortably and plantarflex the ankle. After that, the instructor guided the patient to push her body downwards against the plinth gently. To further enhance patient's relaxation, the instructor guided her to visualize a calming gesture commencing from her eyebrows, ascending through her hairline, continuing over her crown, and culminating at the base of her neck. This visualization further enhanced the relaxation experience. Towards the end of the session, the instructor gently guided her to transition back to a functional state gradually. As the session concluded, the instructor asked the patient to open her eyes and observe her surroundings.

**Figure 1:**
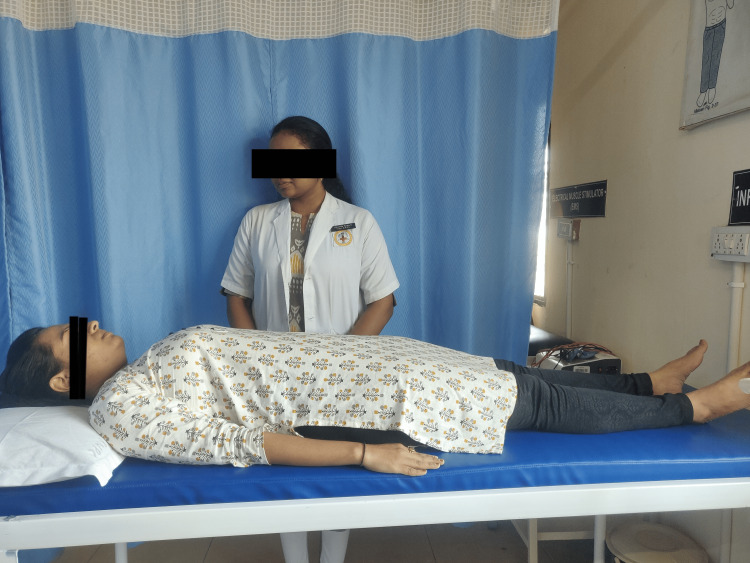
Depicts a patient performing Mitchell's relaxation technique in accordance with the therapist's instructions.

Each session of this relaxation technique lasted for 30 minutes. The session was tailored according to the patient's needs. The instructor carefully designed the process to promote the patient's profound sense of relaxation and tranquillity. The patient is shown executing Mitchell's relaxation technique in Figure 1, precisely as instructed by the therapist.

Group B: Dr. Herbert Benson’s Relaxation Response

**Figure 2:**
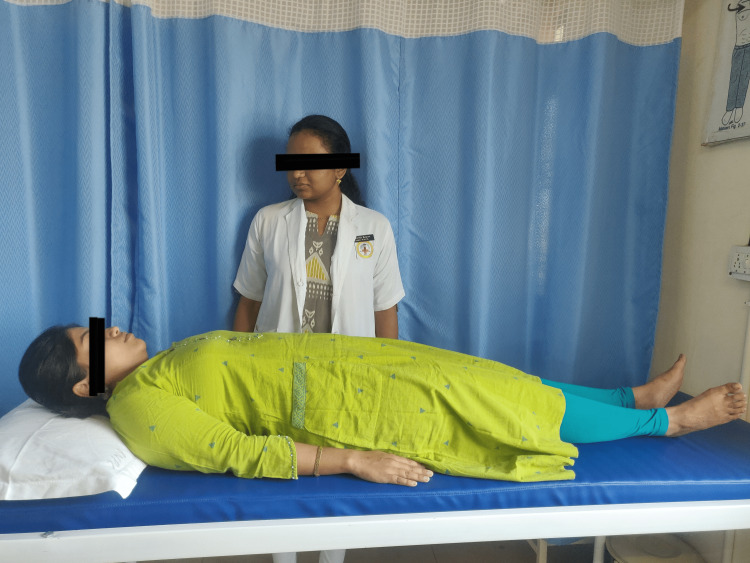
Depicts a patient performing Benson's relaxation technique in accordance with the therapist's instructions.

The therapist individually instructed each subject in Benson's relaxation technique. The patients practised relaxation for a month. Then the therapist asked them to close their eyes. The therapist then recommended that they relax every muscle in their body, starting with their toes and working their way up to their faces. The therapist instructed the patient to focus on relaxing and made aware of their breathing. The therapist recommended the subjects breathe in through their nose and out through their mouth. They were required to practice this breathing exercise every day for 15 to 20 minutes. In addition, the therapist provided clear instructions to the subjects, directing them to dismiss any unsettling thoughts that might arise during the relaxation sessions. After the treatment, the therapist instructed the subjects to sustain the posture briefly (one to two minutes) until the session concluded. To ensure compliance, the therapist made calls and provided daily prompts to the patients to employ the approach. Additionally, other family members assisted in surreptitiously observing the patients while they practised the technique. In Figure 2, a patient is shown executing Benson's relaxation technique with utmost precision, adhering to the therapist's instructions.

Outcomes

Numeric pain rating scale (NPRS): The NPRS is a self-reported measuring instrument consisting of a numerical point scale with extreme anchors of 'no pain' to 'severe pain'. Typically, the scale falls between 0 and 10. Reliability, the extent to which an instrument produces error-free and consistent measurements, holds crucial significance. Studies have shown that the NPRS exhibits moderate to high test-retest reliability, with coefficients ranging from 0.67 to 0.96. Additionally, the NPRS allows for drawing meaningful inferences from the obtained measurement results [19].

Premenstrual syndrome questionnaire: The Premenstrual Syndrome Questionnaire comprises 11 premenstrual symptoms questions and three functional impairment items, considered condensed versions of the scales. The questionnaire demonstrated high reliability with a Cronbach's alpha value of 0.93. To evaluate the structural validity, researchers conducted a confirmatory factor analysis and found that the one-factor and the two-factor models fit the data well [20].

## Results

Table 1 shows the comparison of Group A and Group B concerning mean age. The table presents the statistical analysis of Group A, and Group B. Group A has a mean value of 25.49 with a standard deviation of 3.97. In contrast, Group B has a slightly lower mean of 24.74 and a standard deviation of 4.54. These values represent the central tendency and variability of the data within each group. From the data, Group A tends to have slightly higher average values than Group B. However, it is essential to note that the standard deviation indicates that there is more variation within Group B's data compared to Group A.

**Table 1:** Shows the comparison of Group A and Group B concerning mean age. SD: standard deviation

Group	Mean	SD
Group A	25.4857	3.9740
Group B	24.7429	4.5440

Table 2 shows the comparison of Group A pre- and post-test results concerning PMS Questionnaire using paired t-test. The table displays the descriptive statistics for Group A in a pre-test and post-test scenario. In the pre-test phase, the group's mean score was 69.49, with a standard deviation of 6.97 and a standard error of the mean (SEM) of 1.18. The researchers based the pre-test data on a sample size (N) of 35 participants. Following the intervention or treatment, the researchers conducted the post-test, and the results indicated a decrease in the mean score to 64.26. The post-test also showed a reduced standard deviation of 4.85 and an SEM of 0.82. Like the pre-test, the post-test data had a sample size (N) of 35 participants. The comparison between the pre-test and post-test data demonstrates the impact of the intervention on Group A's performance. The decrease in the mean score suggests a potential change in the participants' performance after the intervention, and the reduction in standard deviation and standard error indicates increased consistency and precision in the results.

**Table 2:** Comparison of Group A pre- and post-test results concerning Premenstrual Syndrome (PMS) Questionnaire using paired t-test. SD: standard deviation, SEM: standard error of the mean

Group A	Mean	SD	SEM	N
Pre-test	69.49	6.97	1.18	35
Post-test	64.26	4.85	0.82	35

Table 3 shows the comparison of Group B pre- and post-test results concerning PMS Questionnaire using paired t-test. The table presents the descriptive statistics for Group B in a pre-test and post-test scenario. In the pre-test phase, the group's mean score was 69.94, with a standard deviation of 9.26 and an SEM of 1.57. The researchers based the pre-test data on a sample size (N) of 35 participants. Following the intervention or treatment, they conducted the post-test, and the results revealed a significant decrease in the mean score to 40.29. The post-test also showed a lower standard deviation of 5.68 and an SEM of 0.96. Like the pre-test, the post-test data had a sample size (N) of 35 participants. The comparison between the pre-test and post-test data demonstrates a substantial change in Group B's performance after the intervention. The significant decrease in the mean score indicates a considerable impact of the intervention on the participants' performance. Additionally, the reduction in standard deviation and standard error suggests increased consistency and precision in the post-test results compared to the pre-test.

**Table 3:** Comparison of Group B pre- and post-test results concerning Premenstrual Syndrome (PMS) Questionnaire using paired t-test. SD: standard deviation, SEM: standard error of the mean

Group B	Mean	SD	SEM	N
Pre-test	69.94	9.26	1.57	35
Post-test	40.29	5.68	0.96	35

Table 4 shows the comparison of the two study groups (Group A and Group B) for post-test PMS Questionnaire scores by t-test. The table presents the descriptive statistics for Group A and Group B. In Group A, the mean score is 58.34, with a standard deviation of 5.25 and an SEM of 0.89. The researchers collected the data in Group A based on a sample size (N) of 35 participants. On the other hand, in Group B, the mean score is 39.09, with a standard deviation of 6.09 and an SEM of 1.03. The researchers collected the data in Group B from a sample size (N) of 35 participants. The comparison between the two groups' data reveals distinct differences in their performance. Group A has a notably higher mean score than Group B, suggesting that, on average, Group A participants performed better than Group B. Additionally, it is worth noting that the standard deviation and standard error for Group A are smaller than those for Group B.

**Table 4:** Comparison of the two study groups (Group A and Group B) for post-test Premenstrual Syndrome (PMS) Questionnaire scores by t-test. SD: standard deviation, SEM: standard error of the mean

Group	Mean	SD	SEM	N
Group A	58.34	5.25	0.89	35
Group B	39.09	6.09	1.03	35

Table 5 compares Group A pre and post-test results to numerical pain scores using paired t-test. The table displays the descriptive statistics for Group A in a pre-test and post-test scenario. In the pre-test phase, the group's mean score was 7.66, with a standard deviation of 1.03 and an SEM of 0.17. The pre-test data was based on a sample size (N) of 35 participants. The post-test was conducted after the intervention or treatment, and the results showed a decrease in the mean score to 5.86. The post-test also displayed a lower standard deviation of 0.77 and an SEM of 0.13. Like the pre-test, the post-test data had a sample size (N) of 35 participants. The comparison between the pre-test and post-test data indicates a significant change in Group A's performance after the intervention. The decrease in the mean score suggests that the intervention impacted the participants' performance, resulting in lower scores in the post-test compared to the pre-test. Additionally, the reduction in standard deviation and standard error suggests that the post-test data points are more tightly clustered around the mean, indicating increased consistency in the results.

**Table 5:** Compares Group A pre- and post-test results to numerical pain scores using paired t-test. SD: standard deviation, SEM: standard error of the mean

Group A	Mean	SD	SEM	N
Pre-test	7.66	1.03	0.17	35
Post-test	5.86	0.77	0.13	35

Table 6 compares Group B pre- and post-test results for numerical pain scores using paired t-test. The table presents the descriptive statistics for Group B in a pre-test and post-test scenario. In the pre-test phase, the group's mean score was 7.69, with a standard deviation of 1.07 and an SEM of 0.17. The pre-test data was based on a sample size (N) of 35 participants. The post-test results showed a significant decrease in the mean score to 2.63. The post-test also displayed a standard deviation of 1.11 and an SEM of 0.19. Like the pre-test, the post-test data had a sample size (N) of 35 participants. The comparison between the pre-test and post-test data indicates a substantial change in Group B's performance after the intervention. The significant decrease in the mean score suggests that the intervention considerably impacted the participants' performance, resulting in much lower scores in the post-test compared to the pre-test. Additionally, the standard deviation and standard error values indicate the variability and precision of the post-test data, respectively. These findings provide essential insights into the effectiveness of the intervention for Group B. They can serve as a basis for further analysis and decision-making in the context of the studied group. The substantial decrease in the mean score after the intervention highlights the significant effect of the treatment on the participants' performance. The standard deviation and standard error values also help understand the spread and reliability of the post-test data. The intervention notably affected Group B's performance, as reflected in the post-test scores.

**Table 6:** Compares Group B pre- and post-test results for numerical pain scores using paired t-test. SD: standard deviation, SEM: standard error of the mean

Group B	Mean	SD	SEM	N
Pre-test	7.69	1.07	0.17	35
Post-test	2.63	1.11	0.19	35

Table 7 compares the two study groups (Group A and Group B) for post-test NPRS Questionnaire scores by t-test. The table presents the descriptive statistics for Group A and Group B. In Group A, the mean score is 5.17, with a standard deviation of 0.71 and an SEM of 0.12. The data in Group A was based on a sample size (N) of 35 participants. On the other hand, in Group B, the mean score is 2.63, with a higher standard deviation of 1.71 and an SEM of 0.20. Similar to Group A, the data in Group B was also collected from a sample size (N) of 35 participants. The comparison between the two groups' data shows distinct differences in their performance. Group A has a higher mean score than Group B, indicating that, on average, Group A participants performed better than Group B. Moreover, the relatively lower standard deviation and standard error in Group A compared to Group B indicate a more compact clustering of data points around the mean in Group A. Consequently, the mean in Group A is more representative of the group's performance. This suggests that the scores in Group A exhibit less variability and are closer to the mean, providing a more focused and reliable measure of the group's overall performance. On the other hand, Group B displays a higher standard deviation and standard error, signifying a greater spread of data points around the mean. This suggests that the scores in Group B are more dispersed and may deviate further from the mean, resulting in a less precise representation of the group's performance.

**Table 7:** Compares the two study groups (Group A and Group B) for post-test Numeric Pain Rating Scale (NPRS) Questionnaire scores by t-test. SD: standard deviation, SEM: standard error of the mean

Group	Mean	SD	SEM	N
Group A	5.17	0.71	0.12	35
Group B	2.63	1.71	0.20	35

## Discussion

Menstrual symptoms of one form or another are common among women of reproductive age. During the luteal phase, physical, emotional, and behavioural symptoms of PMS significantly interfere with a woman's day-to-day activities. These symptoms typically subside a few days after the onset of her period [21]. While the exact aetiology of PMS remains unknown, it is associated with ovarian hormone levels and the timing of symptom development [22]. PMS symptoms can vary in severity, ranging from minor inconveniences to significantly impacting daily life and work. The primary types of symptoms include behavioural, psychological, and physical manifestations [23]. Affective symptoms may persist for several days to two weeks, with a symptom-free period typically preceding ovulation [24].

There is a growing interest in individualized, holistic, and progressive approaches to managing PMS. The initial step in relieving PMS symptoms involves promoting self-screening and providing instruction and counselling on self-care measures, encompassing lifestyle adjustments, dietary management, and coping mechanisms. Subsequently, non-pharmacological strategies, such as cognitive-behavioural therapy and complementary and alternative medicine treatments, should be considered. If symptoms persist, pharmacological approaches, including hormonal, non-hormonal, and symptomatic therapies, are recommended [25]. Mind-body interventions are often referenced as complementary therapies for managing pain [26].

According to some definitions, relaxation refers to a "state of relative freedom" that "breaks the vicious cycle of both concern and skeletal muscular tension," bringing the participant's mind to a sense of balance and peace. Therefore, experts have developed a suggested action plan for physical and mental processes. Relaxation has gained popularity as an effective pain management technique. Various ideas propose that relaxation reduces pain by dissociating the connection between tension and pain [27].

The Benson relaxation method is highly regarded because of its quick, inexpensive, and easy-to-learn nature. This relaxation technique combines various relaxation response techniques [28]. Herbert Benson emphasizes stress reduction as a crucial aspect of meditation. Researchers have shown that Benson relaxation reduces blood lactate levels, heart rate, breathing rate, and muscle tension, calming the patient. Numerous studies provide evidence of Benson relaxation's efficacy in reducing pain intensity [29].

It is widely acknowledged that Mitchell's physiological relaxation can be beneficial in managing stress. Its foundation lies in the physiological concept of reciprocal inhibition. This relatively straightforward technique is easy to learn, requires little focus, and can be extensively practised at home [30].

This study investigated the effectiveness of two relaxation techniques, Benson's and Mitchell's, in addressing PMS symptoms among women aged 21 to 50. The findings highlighted Benson's relaxation technique as notably efficacious (p<0.01), signifying its potential superiority in symptom alleviation. The daily practice of Benson's technique over a month and its emphasis on relaxation and the mind-body connection likely contributed to its superior performance in managing stress-related symptoms. Furthermore, the controlled breathing intrinsic to Benson's technique impacted the autonomic nervous system and stress hormones, augmenting its therapeutic effect. Participant preference and ease of daily engagement could have bolstered Benson's technique. Consequently, the study underscores the favorable potential of Benson's relaxation technique over Mitchell's approach for proficiently managing PMS symptoms among the specified demographic.

Comparison with other studies

Rambod et al. (2013) examined changes in stress, anxiety, and depression symptoms reported by haemodialysis patients who underwent Benson's relaxation technique. The intervention group engaged in Benson's relaxation training twice daily for 15 minutes over four weeks. Researchers used a scale to evaluate sadness, anxiety, and stress levels before and after the sessions. During the intervention, the stress and anxiety levels of the case group significantly changed, but the mean depression value did not show any change. The authors concluded that patients could experience greater calmness during medical therapy, and reducing stress and anxiety may contribute to a more tranquil state for patients [31]. Fenlon et al. conducted a study to explore the potential of relaxation training in reducing hot flashes among women with primary breast cancer. The researchers provided the participants with a single relaxation training session and instructed them to practice the recordings at home daily for one month. In contrast, the control group did not receive any intervention. The results showed significant reductions in the frequency and intensity of hot flashes over the month, suggesting that relaxation training may effectively manage hot flashes in this population [32].

Nesami et al. studied the effects of the Benson Relaxation Method on individuals with rheumatoid arthritis. They selected 50 patients, one group receiving only medication (control) and the other group receiving both medication and Benson's Relaxation Method (experimental). The experimental group showed noticeable differences in anxiety, depression, and subjective well-being compared to the control group, indicating a potential slowdown in the progression of the illness [33]. In Tehran, Kiyani et al. investigated the effects of Benson's Relaxation Method on stress and hemodynamic markers in patients with acute myocardial infarction. The study involved 60 patients in both the case and control groups diagnosed with acute myocardial infarction. The results revealed statistically significant differences in hemodynamic parameters between the case and control groups (p < 0.05). Notably, Benson's Relaxation Method led to improvements in the hemodynamic parameters [34].

Kwekkeboom and Elfa (2008) comprehensively analysed pain-relieving relaxation methods. In their literature search spanning from 1996 to March 2005, they examined various researchers supporting relaxation treatments. Mild muscle relaxation was frequently recommended, particularly for arthritic pain. The study highlighted the effectiveness of Benson's relaxation intervention and jaw relaxation in reducing postoperative pain [35]. Irvin conducted a study to examine the efficacy of eliciting the relaxation response for treating menopausal hot flashes and associated psychosocial difficulties. The volunteer sample consisted of 33 female participants aged 44 to 66. The study found that daily relaxation response inductions significantly reduced the severity and frequency of hot flashes and the associated psychological symptoms [36]. In 2006, Good et al. conducted a study to determine the benefits of relaxation and music on postoperative pain. Most studies found that relaxation techniques and listening to music reduced emotional and outward signs of pain by 60%. However, the study's limited sample size and lack of random assignment raise concerns about the validity of the research's conclusions [37].

Roykulcharoen and Good conducted a randomized controlled experiment to investigate the role of the relaxation response in reducing postoperative pain. The relaxation group reported decreased post-test emotion and pain discomfort compared to the control group. However, relaxation did not significantly reduce anxiety or opioid consumption over six hours [38]. Esch et al. investigated the potential therapeutic applications of the relaxation response for stress-related disorders. This study aimed to examine the relationship between the relaxation response (RR) and problems brought on by stress. Research has demonstrated that the RR treats various stress-related illnesses and health problems [39]. Ferreira and Kulkarni looked at the effects of relaxation methods on premenstrual headaches and fatigue in a comparative interventional experiment. The researchers divided the participants into two groups, administering Mitchell's relaxation and vision meditation to the other, respectively. The researchers used the Global Fatigue Index (GFI) and the Headache Disability Index (HDI) to compare the degree of fatigue and headaches before and after the intervention. This study discovered that Mitchell's relaxation technique and meditation with visualization successfully alleviated fatigue and headaches in premenstrual syndrome sufferers [40].

In research on university students in Cairo, Sohier et al. aimed to assess how well Mitchell's fundamental physiological relaxation technique worked in treating primary dysmenorrhea patients' pain and anxiety. Each participant received Mitchell's basic physiological relaxation technique for 30 minutes three times a week for four weeks, in addition to their daily activities at home. Mitchell's approach was effective in alleviating the symptoms [41]. In 1991, Jackson et al. investigated the effectiveness of the Mitchell method of fundamental physiological relaxation in reducing muscular tension in women with rheumatoid arthritis. The data showed this strategy minimizes muscular tension and balances the body position chosen while agitated [42].

The study has two notable limitations. Firstly, there was no follow-up conducted after the initial intervention. Follow-up data is necessary to assess the long-term effects of the relaxation technique on the participants' outcomes. Follow-up data could have provided valuable insights into the sustainability of the relaxation response in treating stress-related illnesses and health problems over an extended period. Another limitation is that the study lacked blinding of the investigator. This lack of blinding might introduce bias in data collection and analysis. Blinding the investigator would have reduced the potential for unintentional influence or subjective judgments, thereby enhancing the objectivity and credibility of the study's findings.

To address these limitations, researchers could incorporate follow-up assessments at regular intervals in future research to evaluate the persistence of the relaxation response's benefits. Additionally, employing blinding procedures in the study design could minimize bias and improve the reliability of the results.

## Conclusions

Regarding reducing premenstrual syndrome intensity in young people, Benson's relaxation technique is superior to Mitchell's. Due to their simplicity, low cost, and lack of any unfavourable side effects, both methods can be utilised on patients to improve their menstrual health. They should be engrained as a regular practice to enhance the quality of life. Also, they can be used in clinical settings to lessen symptoms, improve quality of life, and lessen stress.
